# Xylazine-Associated Wounds of the Upper Extremity: Evaluation and Algorithmic Surgical Strategy

**DOI:** 10.1016/j.jhsg.2024.05.003

**Published:** 2024-06-19

**Authors:** Richard Tosti, Bryan A. Hozack, Jacob E. Tulipan, Katherine T. Criner-Woozley, Asif M. Ilyas

**Affiliations:** ∗ Rothman Orthopaedic Institute, Philadelphia, PA; † Thomas Jefferson University Hospital, Philadelphia, PA; ‡ AtlantiCare Regional Medical Center, Atlantic City, NJ; § Cooper University Hospital, Camden, NJ ^‖^Drexel University College of Medicine, Philadelphia, PA; ¶ Rothman Opioid Foundation, Philadelphia

**Keywords:** Hand, Surgical strategy, Upper extremity, Xylazine, Xylazine-associated wounds

## Abstract

The coadministration of xylazine, a veterinary tranquilizer, with illicit fentanyl has led to severe soft tissue injuries, ranging from superficial irritation to deep tissue necrosis and even bone involvement, because of multifactorial tissue toxicity. Despite its non-opioid nature, xylazine enhances and prolongs the euphoric effects of fentanyl, exacerbating the potential for abuse. The pathogenesis of the tissue damage from xylazine is multifactorial but most akin to a burn from local tissue injury. With illicit opioids increasingly adulterated with xylazine, particularly in urban areas like Philadelphia, the prevalence of associated wounds, especially in the upper extremities, is anticipated to rise. Managing these wounds demands a multidisciplinary approach, with hand surgeons and reconstructive surgeons playing a central role. This review summarizes the historical context, pharmacodynamics, initial evaluation, wound categorization, algorithmic treatment, and expected outcomes of xylazine-associated wounds.

Xylazine (also known as “tranq, tranq dope, Philly dope, sleep-cut, or zombie drug”) is a non-opioid veterinary tranquilizer that has become a common additive with illicit fentanyl and other opioids.^1^ When xylazine is injected with fentanyl, the result can be severe soft tissue injury ranging from superficial irritation to deep tissue necrosis and even bony involvement (Fig. 1). The pathogenesis of the skin changes from xylazine is multifactorial but most akin to a burn from local tissue injury. Theoretical causes for the tissue toxicity from xylazine include injury from the concentrated acidity, the vasoconstrictive effect on local blood vessels, and overall decreased tissue oxygenation from central nervous tissue depression.^2^ However, the enhanced and prolonged euphoric effect xylazine adds to injected fentanyl will likely only increase the potential for abuse.

**Figure 1:**
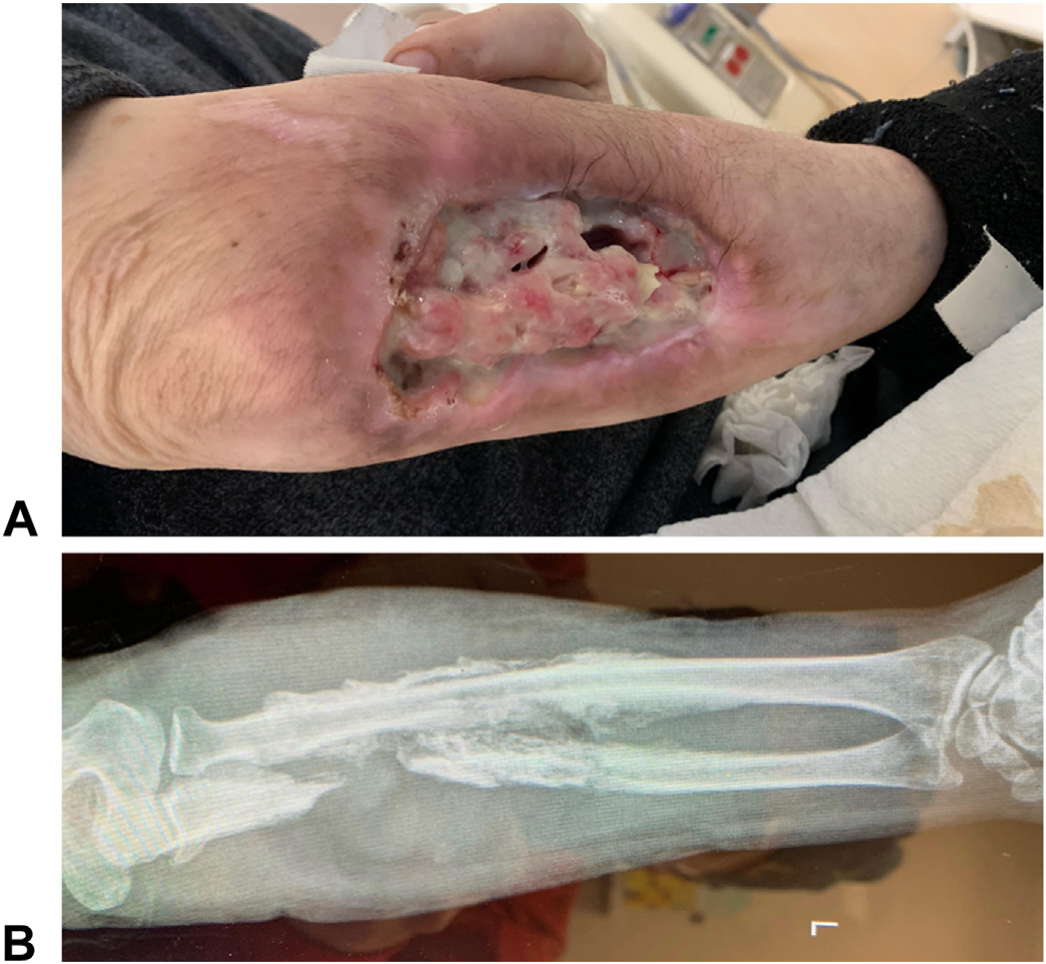
**A** Chronic xylazine-associated wound of the forearm with exposed ulna and secondary osteomyelitis. **B** Shows the X-Ray of the forearm.

The surgical and nonsurgical management of xylazine-associated wounds requires an evidenced-based and multidisciplinary approach. Specifically, skin ulcerations are noted to be six times higher in fentanyl adulterated with xylazine when compared to without xylazine.^3^ Moreover, between 2018 and 2021, the estimated fatal drug poisonings from opioids has increased over 10-fold.^4^ In fact, 90% of the illicit opioid supply in Philadelphia has been reported to be adulterated with xylazine.^2^^,^^5^ As people who inject drugs most often inject into their upper extremities, upper extremity wounds associated with xylazine injection can be anticipated to grow as abuse expands beyond Philadelphia to other urban regions in the US as well as internationally.^6^ As such, hand surgeons and other reconstructive surgeons will be central to effective management.

## Brief History of Xylazine

Originally developed as an anti-hypertensive agent by Farbenfabriken Bayer AG (now Bayer AG) in 1962, xylazine was found to cause severe hypotension and central nervous system depression.^7^^,^^8^ Xylazine was never approved for human use but was instead approved in 1967 as a sedative in large animal anesthesia.^7^^,^^9^ Shortly after its development, xylazine’s danger became quickly evident from multiple reported suicide attempts, accidental injections or ingestions, and illicit production and abuse.^10^, ^11^, ^12^, ^13^

Years later, medical providers in Puerto Rico first reported chronic wound issues in patients using xylazine.^14^ Around this same time, medical examiners in Philadelphia found xylazine in samples of seven patients who had experienced overdose deaths.^15^ More recently, as testing specifically for xylazine has been introduced in certain areas, the prevalence of xylazine has increased and become more widespread throughout the entire US.^16^

## Pharmacodynamics of Xylazine

Xylazine is a non-opioid sedative, which also has analgesic and muscle relaxing properties. It is an alpha-2 adrenergic agonist which is similar in mechanism to clonidine.^17^ When xylazine interacts with central nervous system alpha-2 adrenergic receptors, it causes sedation and bradycardia. Peripherally xylazine interacts with alpha-2 adrenergic receptors on arterioles to cause vasoconstriction. The local interaction on peripheral nerves appears to cause analgesia as well.

## Initial Management

The initial management of a xylazine-associated wound should follow standard advanced cardiac life support protocols if there is any concern for septicemia or cardiopulmonary compromise. Patients found in an obtunded state may additionally require treatment for hypothermia or compartment syndrome. However, although xylazine-associated wounds may be severe and appear concerning for a necrotizing infection, in our experience, xylazine-associated wounds are generally localized and most consistent with a local burn rather than resulting in systemic effects and/or septicemia.

Management of illicit opioid overdose adulterated with xylazine, while outside the scope of this review, is complicated by the fact that xylazine does not respond to naloxone as would be used for most opioid overdoses. Moreover, xylazine intoxication may last up to 8 hours. Yohimbine, an indolealkylamine alkaloid that produces a competitive alpha-adrenergic blockade, has been shown to antagonize the sedative effects of xylazine in animals.^18^ It has been suggested that it could be used in emergency situations for xylazine overdose, but this has not been tested.^19^ Ultimately, fundamental to xylazine intoxication and initial xylazine-associated wound management are supportive care and local wound care, respectively.

## Wound Management

**Figure 2:**
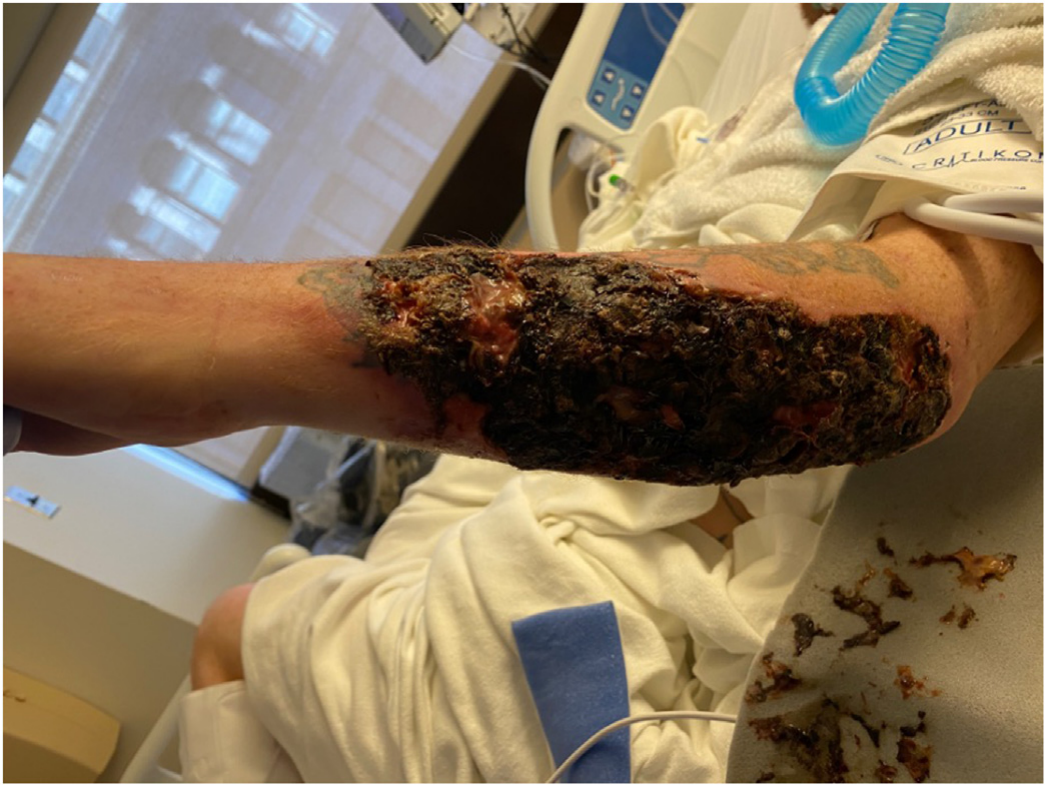
Stage 1 wounds (mild) are characterized as superficial with only partial to full-thickness tissue defect, but without any exposed tendon and muscle, and normal hand function.

**Figure 3:**
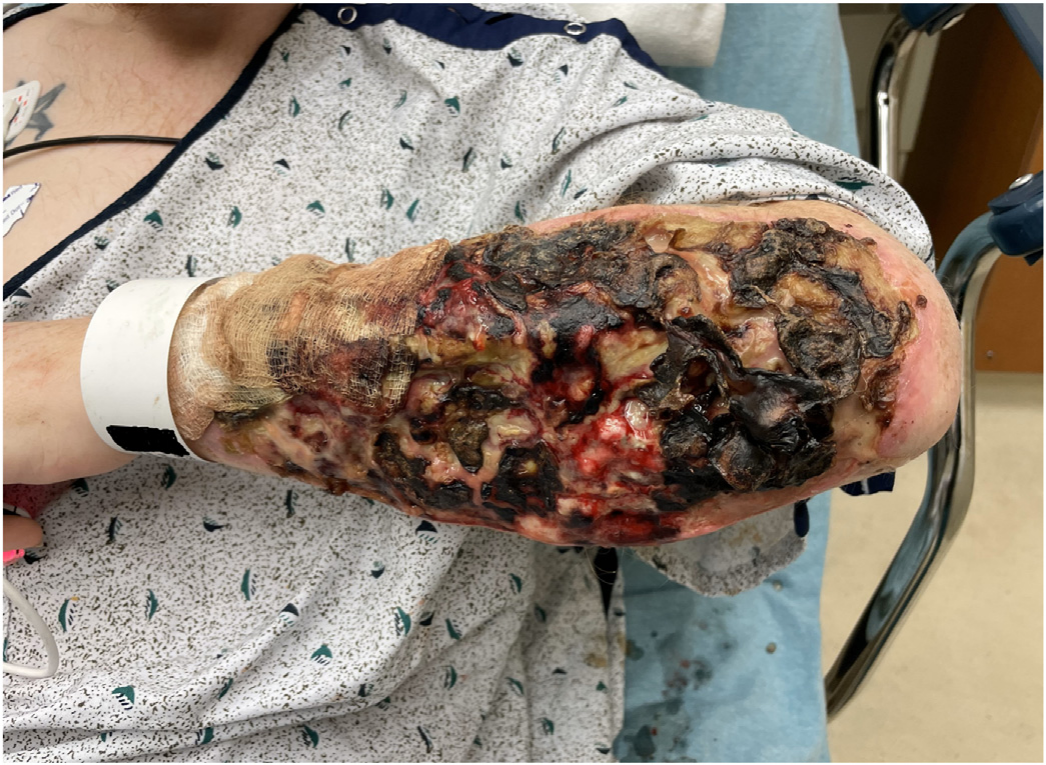
Stage 2 wounds (moderate) are characterized as full-thickness skin loss with exposed and compromised muscle and tendon, but preserved hand function.

**Figure 4:**
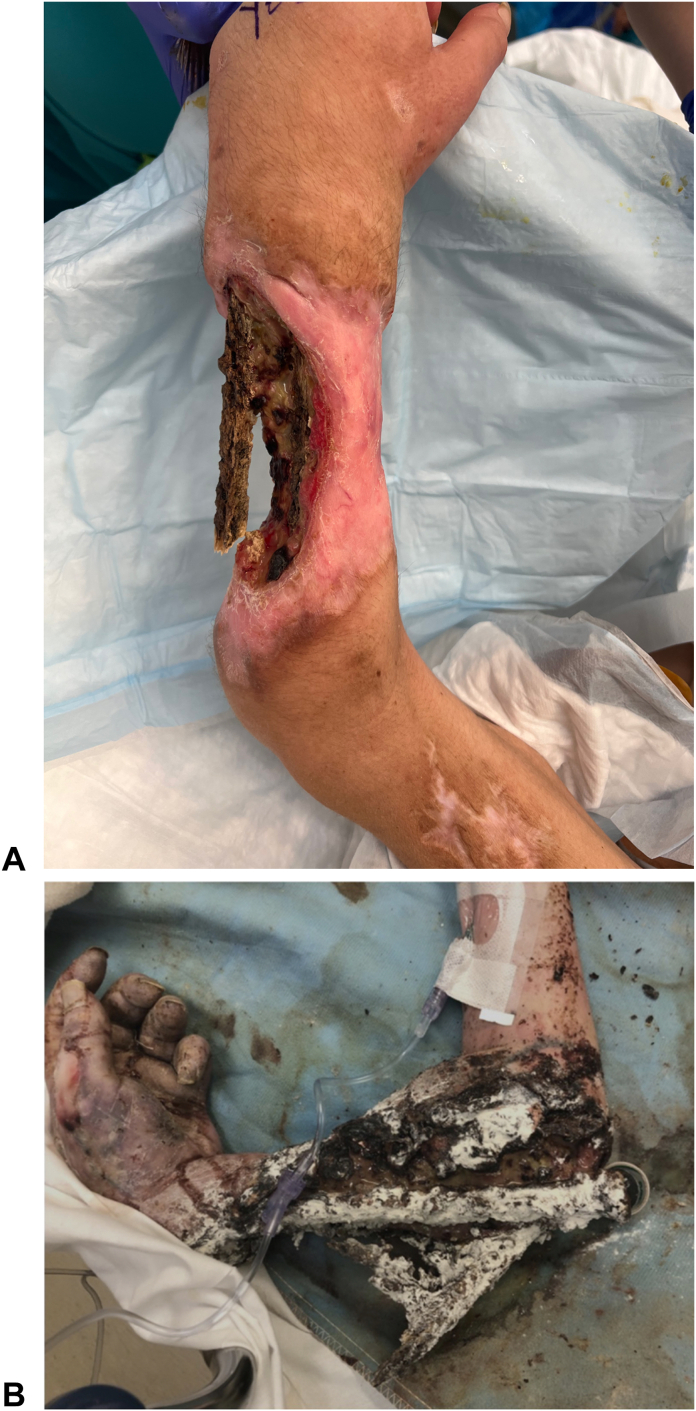
Stage 3 wounds (severe) are characterized by severe muscle necrosis with exposed bone and potentially associated osteomyelitis and/or possible pathological fracture. This stage is subdivided further, with Stage 3A representing preserved hand function and Stage 3B representing lack of hand function. Reprinted with permission from Soderquiest & Solarz et al, JHS Am 2024.^20^

**Figure 5:**
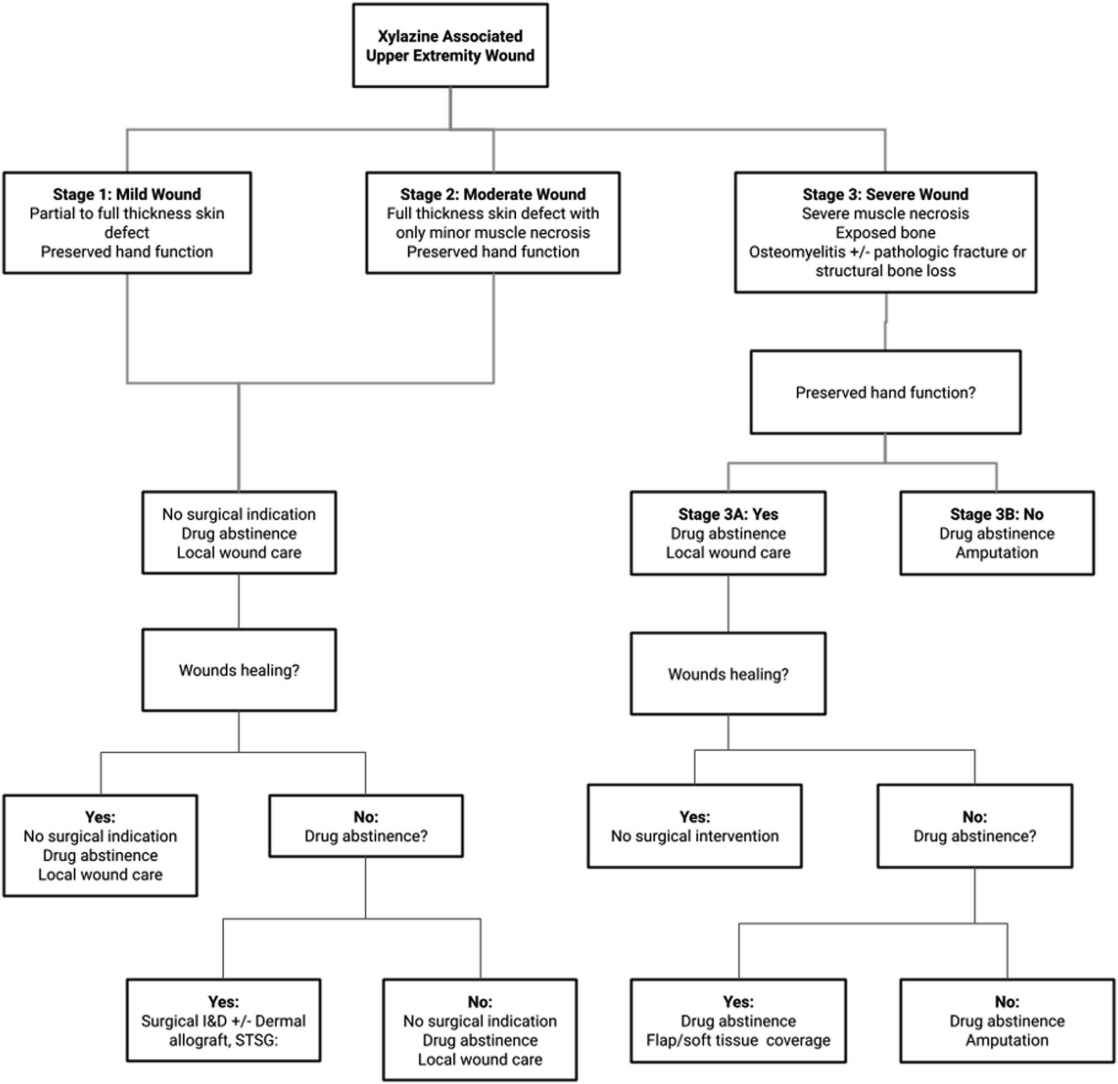
“The Philadelphia Treatment Algorithm for Xylazine Wounds.” I&D, incision and drainage; STSG, split-thickness skin graft.

The combination of local and systemic effects of xylazine leads to local areas of tissue trauma, ischemia, and infection which with cumulative exposures from repeat self-inflected injections result in reticular areas of progressive necrosis (Figure 2, Figure 3, Figure 4). Although often presenting with profound tissue changes, the effects of xylazine on tissue are generally local rather than systemic, and most akin to a burn or local tissue toxicity. In general, xylazine-associated wounds in the upper extremity can be treated with classic reconstructive principles, but given the nature of the self-infliction, the timing and scope of surgical treatment warrants additional considerations. In particular, the incorporation of psychiatry and/or addiction medicine in the treatment of these patients is paramount prior to engagement in heroic surgical interventions. Although unpublished, several of our successful reconstructive efforts have returned months later with graft failures and recurrent wounds because of persistent injections at adjacent sites. Moreover, surgical reconstruction often involves the sacrifice of donor structures, thus in order to limit the depletion of donating structures and to limit massive futile reconstructions, we have developed a treatment algorithm based on our collective experience in Philadelphia (first proposed by co-author, Criner-Woozey KT) recommending a treatment paradigm based on both the physical characteristics of the wound as well as the patient’s addiction status which we have termed “The Philadelphia Treatment Algorithm for Xylazine Wounds” (Fig. 5). This proposed treatment algorithm categorizes wounds into three stages and guides treatment accordingly:

Stage 1 wounds (mild) are characterized as superficial with only partial to full-thickness skin defect, but without any exposed tendon and muscle, and normal hand function (Fig. 2). Stage 2 wounds (moderate) are characterized as full-thickness skin loss with exposed and compromised muscle and tendon, but preserved hand function (Fig. 3). The recommended initial management of Stages 1 and 2 wounds is abstinence from injecting drugs, local wound with good daily hygiene, washing with soap and water, and covering with a sterile gauze. When and if the patient demonstrates drug abstinence and active addiction treatment over 3 months, then surgical intervention can be considered. Specifically, an excisional debridement followed by split-thickness skin grafting is often successful. We prefer to use the anterior thigh as a donor site with 0.012 inch thickness raised from a dermatome. The skin graft is meshed 1.5:1 and spread over the recipient site. Although not absolutely necessary, we generally cover the recipient site with xeroform and a negative pressure wound therapy dressing as a bolster for 5 days.

Stage 3 wounds (severe) are characterized by severe muscle necrosis with exposed bone and potentially associated osteomyelitis and/or possible pathological fracture (Fig. 4). These wounds require an additional level of consideration with preservation of hand function being paramount to determining treatment. Following the “Philadelphia Treatment Algorithm for Xylazine Wounds,” hand function subdivides Stage 3 as 3A and 3B based on hand function being preserved or not, respectively. Unfortunately, with Stage 3B, representing severe tissue and bone compromised without meaningful hand function preserved, amputation is recommended (Fig. 3). We generally recommend that any surgical planning of an amputation involves more than one surgical consultant to concur in the medical records and that the patient can be confirmed able to competently consent to the procedure by the Addiction Medicine or Psychiatry consultants as well, to avoid any medico-legal liability in the future.

In cases of Stage 3A wounds, the patient again must demonstrate drug abstinence and active addiction treatment, prior to considering surgical intervention. When surgical reconstruction is being considered, options include split-thickness skin grafting for exposed muscle or flap coverage for exposed tendon or bone. Flap coverage may be challenging due to peripheral vascular trauma induced either from trauma or infection. Most wounds are on the dorsal hand and forearm but also may extend to the volar forearm. If bone is exposed it is usually the metacarpals dorsally or the ulna. Although an exhaustive review of reconstructive techniques would be outside the scope of this article, the following is a list of coverage options we would consider. For soft tissue defects only: reverse radial forearm flap, posterior interosseous artery flap, groin flap, abdominal perforator flap, anterolateral thigh free-flap. For combined bone and soft tissue defects, common reconstructive options include stage I Masquelet cement spacer and a soft tissue flap followed by subsequent staged bone grafting, or an osteocutaneous free fibula graft which can be used to bridge gaps across the ulna or across the wrist.

As noted earlier and highlighted by the proposed “Philadelphia Treatment Algorithm for Xylazine Wounds,” active addiction will likely lead to further self-inflicted wounds and surgical reconstructive failures. Thus, we believe high level reconstruction should only be performed on patients who are in active treatment and remission from their addiction. Remission from addiction is also difficult to define, but we now advocate waiting 3 months prior to embarking on surgical reconstruction. During this time the patient must demonstrate enrollment and compliance in an anti-addiction program, be abstinent from injecting drugs, and demonstrate good attendance to office visits. In the interim, only local wound care is recommended.

## Expected Outcomes

Overall prognosis in this patient population is poor. Although data on the success of addiction treatment are lacking, the challenge remains obvious. Despite the hurdles to managing the psychological component of opioid addiction, many patients present with xylazine-associated wounds either at a late stage and/or cannot commit to addiction treatment. Although unpublished data, in our experience many patients often refuse to stay for treatment and elopement is very high. In a 2 years case series by Soderquist et al^20^, the authors noted eight patients who presented with exposed radius and ulna and without hand function. All eight patients underwent trans-humeral amputations and one was bilateral.

Xylazine is being used with greater frequency as an adulterant with illicit fentanyl resulting in local tissue trauma, ischemia, and necrosis. The management of xylazine-associated wounds is challenging and involves a multidisciplinary approach. Paramount to successful surgical management is predicated on compliance and success in addiction treatment. If the addiction can be managed, patients can be candidates for reconstructive surgeries. If the addiction cannot be managed, surgical reconstruction should be reserved, assuming the patient is otherwise stable. Unfortunately, demonstrating the power of addiction, patients with xylazine-associated wounds often present late with severe wounds and compromised hand function necessitating amputation.

## Conflicts of Interest

No benefits in any form have been received or will be received related directly to this article.
